# Which first-generation antipsychotics should be “repurposed” for the treatment of schizophrenia

**DOI:** 10.1007/s00406-021-01378-1

**Published:** 2022-01-17

**Authors:** Stefan Leucht, John M. Davis

**Affiliations:** 1 Department of Psychiatry and Psychotherapy, School of Medicine, Technical University of Munich, Munich, Germany; 2 Psychiatric Institute, University of Illinois at Chicago (mc 912), 1601 W. Taylor St, Chicago, IL 60612 USA; 3 Maryland Psychiatric Research Center, Baltimore, MD USA

**Fig. 1:**
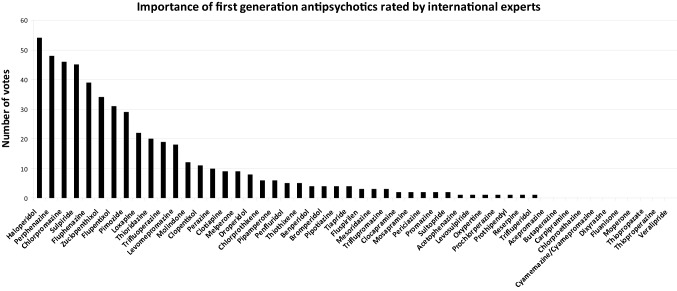
Importance of first-generation antipsychotics according to a survey among 56 international schizophrenia experts, originally used for the choice of drug in Huhn et al. 2019 [1]. The experts were asked to select the 10 first-generation antipsychotics out of the 52 listed by the WHO Collaborating Centre for Drug Statistic which they found most important. The votes were summed up

In a previous editorial for the European Archives of Psychiatry and Clinical Neuroscience, we concluded that most first-generation antipsychotics (FGAs) have been poorly studied in randomized controlled trials (RCTs) so that they should not be the first choices for people with schizophrenia if other affordable options are available. However, we also noted that there are several FGAs which have interesting pharmacological properties. Thus, if their efficacy and safety were better characterized by further randomized controlled trials (RCTs), some of these drugs could turn out to have true clinical advantages and be cost effective. For the network of meta-analysis about the comparative efficacy and safety of antipsychotics for the acute treatment of schizophrenia [1], we had sent a survey to 56 experts in which we asked them to choose 10 of the 52 first-generation antipsychotics listed by the “WHO Collaborating Centre for Drug Statistics” (http://www.whocc.no/atc_ddd_methodology/who_collaborating_centre/) that they found the most important for whatever reason. Counting the votes led to the hierarchy presented in Fig. 1. The survey methods were by far not perfect, but it was useful to guide our choice.

Haloperidol was used as an effective comparator in trials on many FGAs and is therefore one of the best examined antipsychotics. However, the low dose range of haloperidol below 5 mg/day has not been studied well, although a dose–response meta-analysis suggested that its dose–response could be similar to risperidone (maximum efficacious dose in chronic patients approximately 6 mg/day) which could avoid many movement disorders [2].

Chlorpromazine is the other prototypical antipsychotic, but it has not been studied much in the modern trials so that the Cochrane review left many open questions [3] and its side effects may limit the interest in further research.

Clozapine, in a related vein, the prototypical “atypical antipsychotic”, is actually a very old drug developed in Switzerland and Germany in the 1960s, and it is also not well studied. There is only one very small placebo-controlled trial and evidence about key outcomes, such as QTc prolongation and prolactin increase are scarce [1]. Some believe that it is the most effective antipsychotic for negative symptoms, but an appropriate trial in patients with primary negative symptoms is actually lacking [4], and its high risk for sedation and anticholinergic effects speak against this possibility. It is an important drug in psychiatry because of its use in treatment resistant patients and there are many unanswered questions, such as what is its optimal dose [2], should it already be used after the failure of one antipsychotic, etc.

Perphenazine faired very well compared with the second-generation antipsychotics analyzed in the large Clinical Antipsychotic Trials of Intervention Effectiveness (CATIE) study, and it outperformed them in terms of cognition [5]. Therefore, it is an obvious first-generation antipsychotic which should be preserved for treatment and be studied further.

The benzamide sulpiride is interesting because it is the mother drug of amisulpride. It is doubtable whether there would be many differences if the two were compared in an RCT.

Amisulpride generally ranks among the most efficacious antipsychotics in meta-analyses [2]. It was introduced about the same time as risperidone, so it is historically a second-generation drug, but it is an almost pure blocker of the D_2_ family of receptors unlike most other SGAs which are 5HT2_A_ > D_2_ blockers, even though it has some antagonist activity against 5HT7. It was originally developed by a small French company Synthelabo (now merged to SanofiAventis) so that studies in the US that are necessary for FDA approval were not conducted. Moreover, together with cariprazine, it is the only antipsychotic with well-documented efficacy for predominant negative symptoms [4]. Because of its high efficacy, it deserves more investigation, such as whether it should be the drug of first choice for first episode patients and exploring possible mechanisms for its high efficacy.

Zuclopenthixol is available in a short-acting intramuscular formulation which needs to be given every three days which is a good option for some agitated patients. It is also available as an oral and a long-acting injectable formulation.

Flupenthixol binds more tightly to 5-HT_2_ than to dopamine receptors and could therefore have atypical properties. This hypothesis was tested and not really confirmed in one study compared with risperidone, since flupenthixol had higher EPS, but the doses might have been to high (4–12 mg/day) [6].

Loxapine is now available as an inhaler for acutely agitated patients. Therefore, it would be useful to know more about its properties in oral formulations, as it is convenient continue with the same drug.

Molindone seems to decrease weight and could thus be an option in obese patients.

Thioridazine (and pimozide) both increase the QT interval and will therefore probably not be studied much in the future, although thioridazine can be called an old “atypical”, because it causes minimal EPS.

Perazine is a drug which early on replaced chlorpromazine in Germany. It is still used a lot there and in a few other countries such as Poland. According to a large observational study, it produces few movement disorders and could thus fall in the “atypical” category [7]. However, according to a Cochrane review only 7 small studies with 479 participants are available, leaving room for future RCTs [8]. Similarly, melperone has a similar receptor binding profile as clozapine, but it has only been examined in one small trial [9].

Fluspirilene is an interesting butyrophenone, because it is a long-acting injectable which is given weekly. Similarly, penfluridol has been developed as an “oral depot” which needs to be taken only once weekly. It has been withdrawn in the US due to problems in the hematopoietic system, but it is still available in other countries, such as Brazil.

Finally, it should be noted that there are a number of second-generation antipsychotics, namely blonanserine, perospirone, moclapramine, clocapramine, which are mainly used in Japan, and may be useful in other countries.

Our selection of drugs is of course subjective and there may be other candidates. Understandingly, the pharmaceutical industry has little interest in investing old compounds with which they cannot make a profit. However, public funding bodies could be interested, because some old drugs may have clinical as well as economic advantages. That attempts to resuscitate a drug can be successful is exemplified by intravenous droperidol. It was used in many emergency rooms for acutely agitated patients until a black box warning from the Food and Drug Administration (FDA) for dose-related QT prolongation made it difficult to use. However, colleagues in Australia and Hong Kong felt strongly about the importance of this drug. They conducted a series of large RCTs in emergency settings in which droperidol turned out to be highly efficacious and safe, for example [10]. Trials to revive some other first-generation antipsychotics should follow.
